# High In Vitro Antibacterial Activity of Pac-525 against *Porphyromonas gingivalis* Biofilms Cultured on Titanium

**DOI:** 10.1155/2015/909870

**Published:** 2015-01-29

**Authors:** Ji-yin Li, Xue-jin Wang, Li-na Wang, Xiao-xia Ying, Xiang Ren, Hui-ying Liu, Li Xu, Guo-wu Ma

**Affiliations:** ^1^Department of Prosthodontics, School of Stomatology, Dalian Medical University, Dalian 116044, China; ^2^Department of Stomatology, Affiliated Zhongshan Hospital of Dalian University, Dalian 116001, China; ^3^Key Laboratory for Molecular Enzymology and Engineering, The Ministry of Education, College of Life Science, Jilin University, Changchun 130012, China

## Abstract

In order to investigate the potential of short antimicrobial peptides (AMPs) as alternative antibacterial agents during the treatment of peri-implantitis, the cytotoxic activity of three short AMPs, that is, Pac-525, KSL-W, and KSL, was determined using the MTT assay. The antimicrobial activity of these AMPs, ranging in concentration from 0.0039 mg/mL to 0.5 mg/mL, against the predominant planktonic pathogens, including *Streptococcus sanguis, Fusobacterium nucleatum*, and *Porphyromonas gingivalis*, involved in peri-implantitis was investigated. Furthermore, 2-day-old *P. gingivalis* biofilms cultured on titanium surfaces were treated with Pac-525 and subsequently observed and analysed using confocal laser scanning microscopy (CLSM). The average cell proliferation curve indicated that there was no cytotoxicity due to the three short AMPs. The minimum inhibitory concentration and minimum bactericidal concentration values of Pac-525 were 0.0625 mg/mL and 0.125 mg/mL, respectively, for *P. gingivalis* and 0.0078 mg/mL and 0.0156 mg/mL, respectively, for *F. nucleatum*. Using CLSM, we confirmed that compared to 0.1% chlorhexidine, 0.5 mg/mL of Pac-525 caused a significant decrease in biofilm thickness and a decline in the percentage of live bacteria. These data indicate that Pac-525 has unique properties that might make it suitable for the inhibition the growth of pathogenic bacteria around dental implants.

## 1. Introduction

Dental implants have revolutionised dental rehabilitation and prosthetic dentistry and have become a commonly used alternative to conventional removable and fixed partial dentures. The high survival rate reported in several 10-year follow-up studies has led to a widespread acceptance and use of dental implants [1–3]. Although the general impression of implant therapy is that the success rate is high, and inflammation or peri-implantitis is a common occurrence. Peri-implantitis is an inflammatory disease of infectious origin, which ultimately leads to the loss of the bone that supports the implant. It is a biofilm-mediated disease, and the attachment of planktonic pathogenic bacteria to the surface of the dental implant is the initial step in biofilm formation.* Porphyromonas gingivalis* is a late colonizer compared to* Streptococcus sanguis* and* Fusobacterium nucleatum*, which colonize earlier within the biofilm community in the subgingival crevice around dental implants; however,* P. gingivalis* is regarded as the predominant Gram-negative pathogen involved in the initiation and progression of severe forms of peri-implantitis [4, 5]. Thus, effective control of the attachment and proliferation of pathogens, especially* P. gingivalis,* around a dental implant, is key to preventing and/or treating peri-implantitis and improving the survival and success of dental implants.

Natural host defense antimicrobial peptides (AMPs) are small cationic peptides, which are produced by most living organisms, including bacteria, insects, plants, and vertebrates [6]. These peptides are low molecular weight proteins with broad-spectrum antimicrobial activity against many pathogens, including some multidrug-resistant bacteria. An updated database of AMPs is available online at http://aps.unmc.edu/AP/main.php. To date, more than 2,200 AMPs of different origins have been reported. Human saliva and gingival fluid contain at least 45 different AMPs that belong to several different functional classes, ranging from small cationic peptides to enzymes and protein macromolecule. This functional and structural diversity could be necessary to protect the oral epithelia from the large number of potentially harmful microbes, maintain the homeostasis of commensal and pathogenic oral bacteria, and prevent biofilm formation and subsequent gingival inflammation around natural teeth and dental implants. However, several drawbacks of biologically active natural AMPs have been recently described, including systemic and local toxicity; reduced activity based on salt, serum, and pH sensitivity; susceptibility to proteolysis and pharmacokinetic and pharmacodynamic factors; and high manufacturing cost [7, 8]. Appropriately designed peptidomimetics are a possible solution to these problems and have shown low toxicity towards mammalian cells, stability against proteolytic enzymes, and low cost of synthesis [9, 10].

Short AMPs are particularly attractive due to their lower synthesis cost and better structural stability. A novel short Trp-rich peptide, Ac-KWRRWVRWI-NH_2_, designated Pac-525, exhibited improved activity against both bacteria and fungi [11, 12]. Another short peptide, KKVVFKVKFK-NH_2_, designated KSL, was confirmed to have high efficiency against clinically important pathogens in the oral cavity, especially to oral streptococci [13]. Moreover, KSL was reported to inhibit* Streptococcus mutans* biofilm formation [14]. In addition, the KSL analogue, KKVVFWVKFK-NH_2_, designated KSL-W, exhibited excellent stability, adsorption, and sustained release performance and has been used in the chewing gum, as a potential antiplaque agent [15, 16]. As mentioned above, previous studies demonstrated that short AMPs mainly inhibited Gram-positive oral and nonoral bacteria, as well as fungi. However, data regarding the inhibition effects of these short AMPs on peri-implant pathogenic bacterial growth, especially of Gram-negative bacteria, is still unclear. Further, the effect of AMPs on biofilms is also unknown.

In the current study, we investigated the antimicrobial activity of three short AMPs against adherent pathogenic bacteria for planktonic state in vitro, which are indicative of the initial occurrence and development of peri-implantitis, including* S. sanguis, F. nucleatum*, and* P. gingivalis*. Pac-525 exhibited the highest antibacterial activity against all the tested bacteria. Furthermore,* P. gingivalis *biofilms cultured on titanium were treated with Pac-525 and compared to those treated with the conventional antibiotic, DXH, with the aim of providing information for further clinical use.

## 2. Materials and Methods

### 2.1. AMPs Synthesis

Pac-525 (Ac-KWRRWVRWI-NH_2_, molecular weight: 1,426.70 Da), KSL-W (KKVVFWVKFK-NH_2_, molecular weight: 1,307.69 Da), and KSL (KKVVFKVKFK-NH_2_, molecular weight: 1,249.65 Da) were synthesized and used in our study. A high-efficiency solid-phase peptide synthesis was performed by ChinaPeptides Co., Ltd. in Shanghai, China, by using an automatic peptide synthesizer (Symphony; Protein Technologies, Tucson, AZ). The purified peptide was characterised using analytical high performance liquid chromatography (HPLC; Shimadzu, Kyoto, Japan) and mass spectrometry (MS, Finnigan TSQ 7000; Thermo, Waltham, MA) with purity of over 99%. All the synthesized peptides were freeze-dried and stored at −20°C.

### 2.2. Cytotoxicity Assay

A MTT (3-[4,5-dimethylthiazol-2-yl]-2,5-diphenyltetrazolium bromide) assay was performed using MG-63 cells to assess the cytotoxicity of the AMPs. MG-63 cells were provided by Peking University and cultured in Dulbecco's modified Eagle's medium (DMEM; Gibco, New York City, NY) supplemented with 10% fetal bovine serum (FBS; Gibco), penicillin (100 IU/mL; Gibco), and streptomycin (100 *μ*g/mL; Gibco) at 37°C in a humidified 5% CO_2_ incubator. We cultured 5 × 10^3^ MG-63 cells in 96-well plates and allowed them to grow to confluence. The cell culture medium was then replaced by twofold serial dilutions of AMPs, with final concentrations of Pac-525, KSL-W, and KSL peptide ranging from 0.0039 mg/mL to 0.5 mg/mL. DMEM without AMP served as a negative control. After 24 h of culture, 20 *μ*L MTT dye (5 mg/mL in PBS) was added to each well and incubated at 37°C for 4 hours in the dark. During this process, metabolically active cells reduced the MTT to purple formazan. Following incubation, MTT was aspirated from each well, and 150 *μ*L of dimethyl sulfoxide (DMSO) was added to solubilize the formazan crystals; the plate was then incubated for 20 min at room temperature in the dark. The optical density (OD) values for each well were measured at 490 nm by using a microplate reader (Thermo Fisher Scientific, Waltham, MA). A higher absorbance is related to a higher formazan concentration, which indicates higher metabolic activity of MG-63 cells in the wells.

### 2.3. Bacterial Strains and Growth Conditions

*S. sanguis* ATCC10556,* F. nucleatum* ATCC 10953, and* P. gingivalis* ATCC 33277 were used in the study. Strains were kindly provided by the School of Stomatology, Capital Medical University (Beijing, China).* F. nucleatum* was cultured in a liquid Bacto-tryptone-yeast extract-ascorbic acid-glucose 5 mg/mL NaCl solution;* P. gingivalis* and* S. sanguis* were cultured on brain-heart infusion (BHI, Difco; USA) media supplemented with hemin (5 *μ*g/mL; Sigma) and vitamin K1 (1 *μ*g/mL; Sigma). All cultures were incubated at 37°C under anaerobic conditions (80% N_2_, 10% CO_2_, and 10% H_2_). Bacterial cells were harvested by centrifugation at 10,000 × g for 10 min, washed with sterile phosphate-buffered saline (PBS), diluted to 1 × 10^8^ CFU/mL by using a spectrophotometer-based standard curve calculation, and stored at −20°C.

### 2.4. Bacterial Susceptibility Assay

The minimum inhibitory concentration (MIC) and minimum bactericidal concentration (MBC) were measured according to Clinical and Laboratory Standards Institute (CLSI) guidelines [17]. Briefly, for MIC determination, twofold serial dilutions of AMPs were prepared in BHI medium and the final concentration ranged from 0.0039 mg/mL to 0.5 mg/mL. Cultures of* S. sanguis*,* F. nucleatum*, and* P. gingivalis *were adjusted to 2 × 10^6^ CFU/mL with BHI, and 100 *μ*L of inoculum was added to each well of 96-well flat-bottom microtitre plates (Costar 3599; Corning Inc., Corning, NY) containing 100 *μ*L of a series of dilutions of Pac-525, KSL-W, and KSL peptides. BHI with 1 × 10^6^ CFU/mL bacterial suspension without AMP served as a negative control. Chlorhexidine (CHX) at 0.1% served as a positive control. The wells were examined for turbidity after incubation at 37°C under anaerobic conditions for 48 h, referenced by the negative and positive control wells. MIC was defined as the endpoint (the well with the lowest concentration of AMPs) at which no turbidity could be detected with respect to controls. The absorbance at 620 nm (A620) was measured using a microplate reader (Thermo, Waltham, MA) to assess bacterial cell growth. To measure the MBC, an aliquot of 20 *μ*L cell suspension was taken from each clear well containing Pac-525, KSL-W, and KSL peptides, at a concentration above the MIC, and bacterial cells were enumerated after incubation at 37°C under anaerobic conditions for 48 h. The MBC was determined as the lowest AMP concentration that produced no visible colonies on the surface of an agar plate. The tests were performed in triplicate.

### 2.5. Effects of Pac-525 Peptide on* P. gingivalis* Biofilm Cultured on Titanium

#### 2.5.1. Biofilm Formation of* P. gingivalis* on Titanium Discs

**Figure 1:**
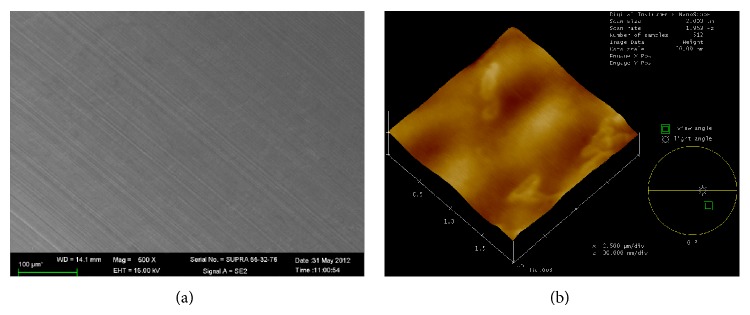
Physical morphology of titanium. (a) SEM image; (b) AF image.

Commercially pure titanium discs (10 mm in diameter and 1 mm in thickness) were polished using waterproof silicon carbide paper until 2,400 c, and morphological images were captured using a Scanning Electron Microscope (SEM, QUANTA 450; SA) and an Atomic Force Microscope (AF, Nanoscope IIIA^+^; Veeco, USA) and shown in Figure 1. The titanium surface had a smooth and uniform physical structure with a roughness of 0.027 *μ*m, which corresponds to the features of the smooth neck of the clinical dental implants [18]. To form an acquired salivary pellicle on titanium and to prepare for bacteria culture, we coated the discs with 300 *μ*L of unstimulated human saliva at 37°C for 4 h, washed them twice with PBS, and transferred them into 24-well plates.* P. gingivalis* biofilms were developed using a modified version of the method described by Noiri et al. [19]. Briefly, 2 mL BHIS with 1 × 10^6^ CFU/mL bacterial suspension of* P. gingivalis* was cultured on the surface of titanium discs and aerobically incubated at 37°C for 48 h. To observe the formation and morphology of* P. gingivalis* cells on the titanium surface, the titanium discs were gently rinsed twice with PBS, fixed with 2.5% glutaraldehyde in 0.1 M cacodylic acid buffer overnight, sequentially dehydrated in a series of ethanol (20%, 50%, 70%, 90%, and 100%) for 10 min each, immersed in isoamyl acetate for 1.5 min, sputter-coated with a thin layer of Au-Pd, and observed and imaged using SEM.

#### 2.5.2. Live/Dead Staining for Biofilms Treated with Pac-525

Following* P. gingivalis* biofilm formation, each titanium disc with 2-day-old biofilm was washed three times with PBS and transferred into a new 24-well plate. BHI containing Pac-525 peptide at concentrations of 0.125 mg/mL, 0.25 mg/mL, and 0.5 mg/mL (equal to the multiple proportions of MBC) was added into the wells and was further aerobically incubated at 37°C for 24 h. BHI without Pac-525 peptide served as a negative control, and 0.1% CHX served as a positive control. To ascertain the viability of* P. gingivalis* biofilms, cells were stained using a LIVE/DEAD BacLight Bacterial Viability Kit (L-13152; Molecular Probes Inc., Eugene, OR) for 20 min in the dark. Live bacteria were stained with SYTO 9 to emit a green fluorescence signal, and dead bacteria were stained with propidium iodide to produce red fluorescence. Confocal laser scanning microscopy (CLSM, Leica TCS SP2; Leica Microsystems, Germany) with beam path settings (488 nm and 543 nm) was used to observe the stained biofilm on the titanium discs. Three independent biofilm experiments were performed, and at least three image stacks per experiment were collected.

### 2.6. Statistical Analysis

Statistical analyses were performed using the SPSS 17.0 package. For the cell proliferation curve, OD_490_ values were compared using the* t*-test for independent samples. In the evaluation of biofilm formation, biofilm thickness and live bacteria percentage of* P. gingivalis* were compared using one-way analysis of variance (ANOVA). A *P* value of <0.05 was considered statistically significant.

## 3. Results

### 3.1. MTT Analysis of MG-63 Cells

**Figure 2:**
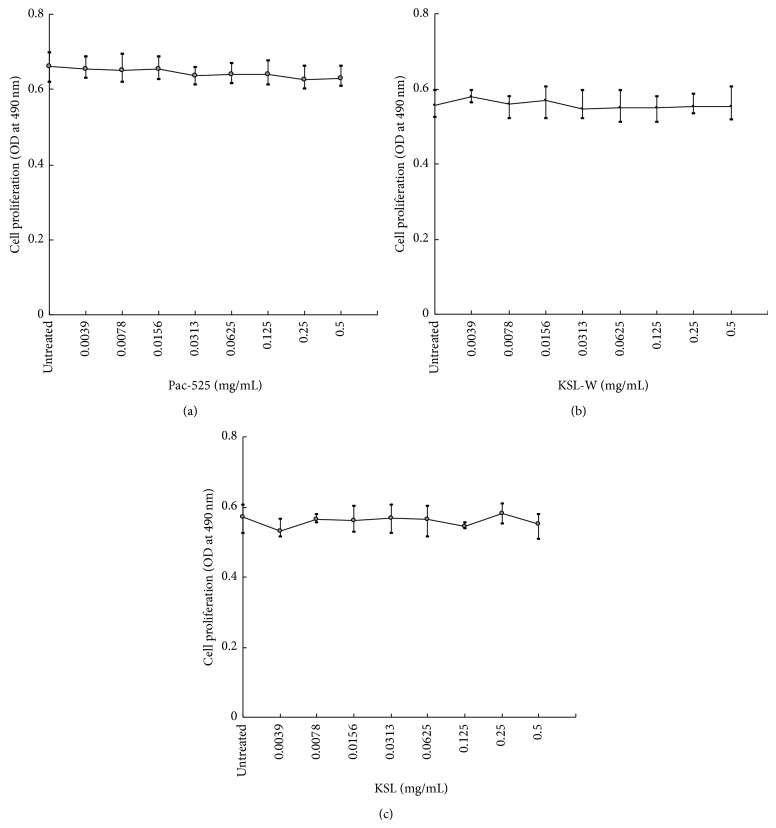
Proliferation curves for MG-63 cells exposed to (a) Pac-525, (b) KSL-W, or (c) KSL antimicrobial peptide solutions at concentrations ranging from 0 to 0.5 mg/mL.

Figure 2 shows the average cell proliferation curve, with the concentration of AMPs on the *X*-axis and OD values on the *Y*-axis. Compared with the untreated group, the absorbance values for each well (treated with Pac-525, KSL-W, or KSL) were similar for each concentration. There was no statistical difference between the AMP-treated group and the untreated group and no statistical difference among the eight concentrations of AMPs.

### 3.2. Minimum Inhibitory Concentrations and Minimum Bactericidal Concentrations of AMPs against* S. sanguis*,* F. nucleatum*, and* P. gingivalis*

MIC and MBC recorded for the three antibacterial peptides against planktonic bacteria of* S. sanguis*,* F. nucleatum*, and* P. gingivalis* are summarized in Table 1. Amongst the tested bacterial strains,* S. sanguis* was susceptible to Pac-525, with a MIC value of 0.125 mg/mL and a MBC value of 0.5 mg/mL, whereas it was insensitive to KSL-W and KSL within the range of tested concentrations. The MIC value of Pac-525 was the lowest: 0.0625 mg/mL for* P. gingivalis* and 0.0078 mg/mL for* F. nucleatum*. Similarly, the MBC value of Pac-525 was the lowest, with 0.125 mg/mL for* P. gingivalis*, and 0.0156 mg/mL for* F. nucleatum*.

### 3.3. The Effects of Pac-525 Peptide on* P. gingivalis* Biofilm

**Figure 3:**
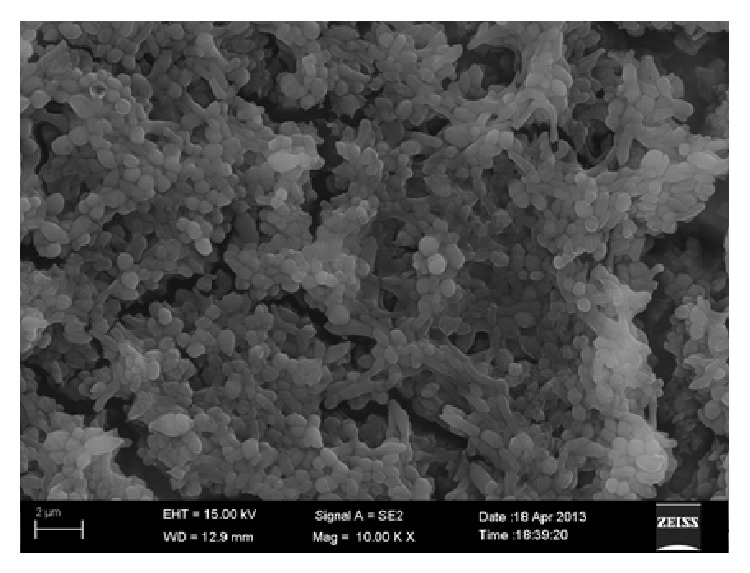
SEM image of a* P. gingivalis* biofilm on titanium.

*P. gingivalis *suspension formed an intact mature biofilm on the titanium surfaces after anaerobic incubation for 48 h. The bacterial cells in the biofilm were uniformly distributed, closely packed, and had channels connecting each other (Figure 3).

**Figure 4:**
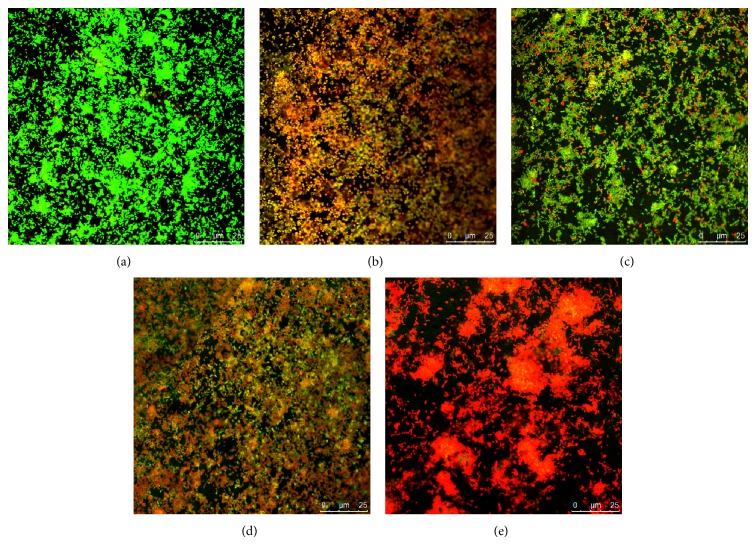
Confocal laser scanning microscopy images of* P. gingivalis* biofilms on titanium discs (oil, ×63). (a) Untreated* P. gingivalis* biofilm (control); (b)* P. gingivalis* biofilm treated with 0.1% chlorhexidine (CHX); (c)* P. gingivalis* biofilm treated with 0.125 mg/mL Pac-525; (d)* P. gingivalis* biofilm treated with 0.25 mg/mL Pac-525; and (e)* P. gingivalis* biofilm treated with 0.5 mg/mL Pac-525.

**Figure 5:**
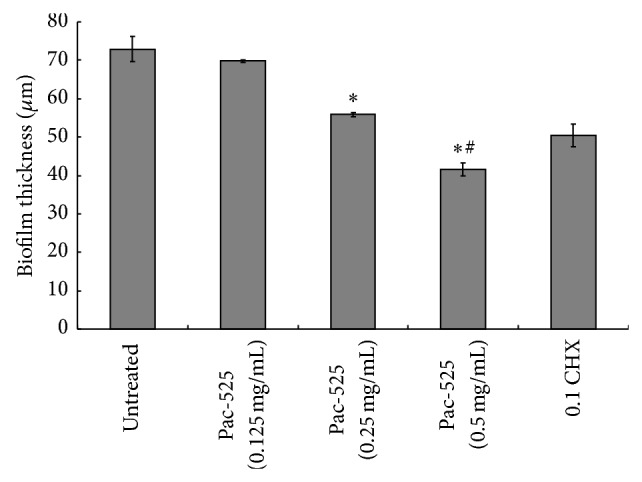
Average thickness of* P. gingivalis* biofilms (on titanium discs) treated with Pac-525. ^*^Significantly different from untreated control. ^#^Significantly different from CHX-treated control (*P* < 0.05).

**Figure 6:**
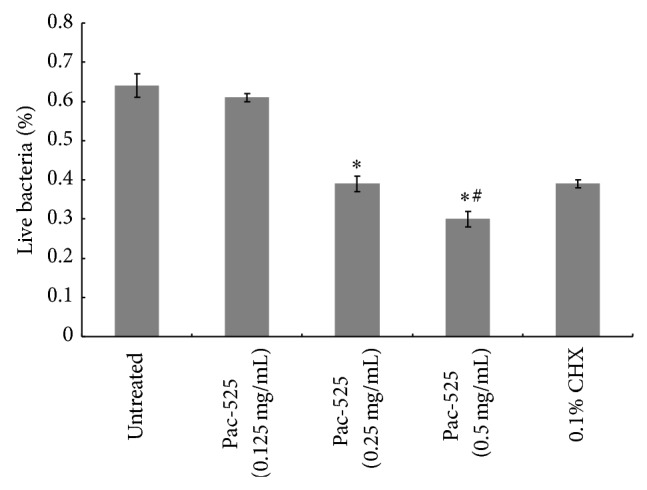
Percentage of live bacteria in* P. gingivalis* biofilms (on titanium discs) treated with Pac-525. ^*^Significantly different from untreated control. ^#^Significantly different from CHX-treated control (*P* < 0.05).

*P. gingivalis* biofilms on the surface of titanium discs were imaged by CLSM and are shown in Figure 4. In the images, the live bacteria were stained and emitted a green fluorescence signal, while the dead one was stained and produced red fluorescence. The nontreated control biofilm emitted green fluorescence (a), and the CHX-treated biofilm emitted red (b). Compared to the controls, Pac-525 had a significant impact on the viability of biofilm cells. The biofilm showed extensive green fluorescence and scattered red fluorescence after treatment with 0.125 mg/mL Pac-525 (c), obvious red fluorescence and inconspicuous green fluorescence with 0.25 mg/mL Pac-525 (d), and almost completely red fluorescence with 0.5 mg/mL Pac-525 (e). The average thickness of* P. gingivalis* biofilms was 55.89 *μ*m and 41.64 *μ*m at Pac-525 concentrations of 0.25 mg/mL and 0.5 mg/mL, respectively, which were significantly lower than those in the nontreated control group (*P* < 0.05). Furthermore, the thickness of biofilms treated with 0.5 mg/mL Pac-525 was significantly lower than that in the CHX-treated control group (*P* < 0.05, Figure 5). Similar to the results for biofilm thickness, live bacteria percentages were 0.39 ± 0.02 and 0.30 ± 0.02 after treatment with Pac-525 concentrations of 0.25 mg/mL and 0.5 mg/mL, respectively. These values were significantly lower than those of the nontreated control group (*P* < 0.05), whereas the live bacteria percentage of the 0.5 mg/mL Pac-525-treated group was significantly lower than that of the CHX-treated control group (*P* < 0.05, Figure 6), suggesting that Pac-525, especially at a concentration of 0.5 mg/mL, had the favourable ability to decrease biofilm thickness, diffuse cell distribution, and reduce* P. gingivalis* viability. These results indicated that Pac-525 was not only able to kill* P. gingivalis* within the biofilm on the titanium but was also able to cause the mature biofilm to disintegrate.

## 4. Discussion

CHX, a membrane active bisbiguanide, is regarded as the “gold standard” antibacterial agent in oral care for reducing plaque and biofilm formation and thus preventing caries, gingivitis, periodontitis, and peri-implantitis. However, long term use of CHX in clinical application can be contraindicated due to oral mucosa irritation, the risk of tooth staining, and dental calculus formation [20]. Thus, it is important to develop alternative antimicrobial agents with fewer side effects. AMPs, such as Pac-525, KSL-W, and KSL, have been reported to efficiently protect the host from bacterial or fungal pathogens and to kill or inhibit cells from planktonic and biofilm cultures; thus, they have been proposed as promising alternative antimicrobial agents in the treatment of oral disease. In this study, we tested the antibacterial activity of these AMPs against the predominant pathogenic bacteria involved in peri-implantitis to determine the most effective AMPs for use as antibacterial agents for dental implants.

One of the most desired properties of AMPs is low toxicity to eukaryotic cells. In this study, an MTT assay was conducted with MG-63 cells to assess cytotoxicity. The three short AMPs did not exhibit cytotoxic effects, which was consistent with previous results that examined the proliferation of keratinocyte cells and human gingival fibroblasts [13, 21, 22]. Our findings confirmed that all the tested short AMPs had high biosafety and were suitable for use as antimicrobial agents in the oral cavity.

Amongst the bacterial strains tested under clinical conditions of dental implants,* S. sanguis* is the early colonizer and* P. gingivalis* is the late one.* F. nucleatum* is the major coaggregation bridge organism that links early and late colonizers. In this study,* S. sanguis* appeared insensitive to KSL-W and KSL within the range of tested concentrations, which differed from the results of Concannon et al. [13] and Liu et al. [14]. This variation may be due to differences in AMPs purity, bacterial strains, and concentrations of bacterial suspension and AMPs solution used in each experiment. However,* S. sanguis* was susceptible to Pac-525, with a MIC value of 0.125 mg/mL and a MBC value of 0.5 mg/mL. For* P. gingivalis* and* F. nucleatum*, the MIC values of Pac-525 were 0.0625 mg/mL and 0.0078 mg/mL, respectively, which were half of the values of KSL and KSL-W. Therefore, among the three short AMPs, Pac-525 was the most efficient in inhibiting the tested planktonic bacteria. To our knowledge, this is the first report describing the inhibitory effects of Pac-525 against planktonic* S. sanguis*,* F. nucleatum*, and* P. gingivalis*. Our findings suggest that Pac-525 has the ability to prevent the adherence of bacteria to the surface of dental implants and therefore decrease biofilm formation, thus decreasing the occurrence to peri-implantitis.

Biofilms have been shown to be many times more resistant to antibiotics and more toxicity to host than planktonic or free-swimming cells so as to improve the possibility of metabolization and survival in the antibacterial conditions [23, 24]. Bacteria that live inside a biofilm are strongly resistant to antimicrobials and exhibit little sensitivity to host defense systems [25]. Because of* P. gingivalis* being the predominant pathogenic bacteria involved in peri-implantitis, we studied the effects of the Pac-525 peptide on* P. gingivalis* biofilms, based on MIC and MBC results suggesting that Pac-525 inhibits planktonic bacteria. Furthermore, commercially pure titanium, having a roughness similar to that of clinical implants, was selected as the carrier material for* P. gingivalis *biofilm formation, in order to imitate clinical conditions and interaction between* P. gingivalis *biofilms and dental implants. The results showed that when treated with 0.5 mg/mL Pac-525, the biofilm thickness, and the percentage of live bacteria were the lowest among the tested groups and were even significantly lower than those of the groups treated with CHX. This demonstrated that Pac-525 has the ability to kill bacteria within* P. gingivalis* biofilms cultured on a titanium surface. Data from our investigation indicated that the effective inhibitory concentration of Pac-525 against* P. gingivalis *biofilm was eight times higher than the MIC for planktonic cells, whereas previous studies showed that inhibitory concentrations against biofilms are often 10 to 1,000 times higher than MICs for planktonic cells [26–28]. Hence, compared to a conventional antibacterial agent, Pac-525 had the remarkable advantage of inhibiting* P. gingivalis *biofilm formation. The inhibitory effect might be related to the structure of Pac-525, which is a novel Trp-rich peptide containing nine amino acids. Trp has a high potential for insertion into membranes and likely endows the Trp-rich Pac-525 peptide with the ability to enter membranes and to affect the lipopolysaccharides of Gram-negative bacteria [29]. Furthermore, Li et al. [30] reported that a derivative of D-Nal-Pac-525 was designed by replacing the tryptophans of Pac-525 with D-*β*-naphthyalanines and was proved to have superior antibacterial activities against the planktonic* S. Mutans* and its biofilm. This report suggests that both Pac-525 and its derivative are potential candidates to prevent oral diseases in terms of dental caries, periodontitis, and peri-implantitis.

In conclusion, the present study demonstrated that Pac-525, KSL-W, and KSL were not cytotoxic and showed high biosafety. Meanwhile, the AMPs showed favourable inhibitory effects against the predominant planktonic pathogenic bacteria involved in peri-implantitis, including* S. sanguis*,* F. nucleatum*, and* P. gingivalis*, although* S. sanguis* appeared insensitive to KSL-W and KSL within the range of tested concentrations. Among the three short AMPs tested, Pac-525 was the most efficient inhibitor of planktonic bacteria. Furthermore, Pac-525 was confirmed to have the ability to kill bacteria within the* P. gingivalis* biofilm cultured on the titanium surface. Thus, the short AMPs tested in our study had unique properties and could have a potential clinical application in the prevention and/or treatment of peri-implantitis by the inhibition of pathogenic bacteria around dental implants.

## Figures and Tables

**Table 1:** Susceptibility of *S*. *sanguis*, *F*. *nucleatum*, and *P*. *gingivalis* to AMPs in vitro.

Bacterial strain	AMPs					
	AMPs concentration (mg/mL)^a^					
	Pac-525		KSL-W		KSL	
	MIC	MBC	MIC	MBC	MIC	MBC
*S*. *sanguis* 10556	0.125	0.5	—	—	—	—
*F*. *nucleatum* 10953	0.0078	0.0156	0.0156	0.0313	0.0156	0.0313
*P*. *gingivalis* 33277	0.0625	0.125	0.125	0.5	0.125	0.5

^a^Values represent the geometric mean of three individual experiments.
